# Primary Chicken and Duck Endothelial Cells Display a Differential Response to Infection with Highly Pathogenic Avian Influenza Virus

**DOI:** 10.3390/genes12060901

**Published:** 2021-06-10

**Authors:** Zhen Wei Marcus Tong, Anjana C. Karawita, Colin Kern, Huaijun Zhou, Jane E. Sinclair, Limin Yan, Keng Yih Chew, Sue Lowther, Lee Trinidad, Arjun Challagulla, Karel A. Schat, Michelle L. Baker, Kirsty R. Short

**Affiliations:** 1 School of Chemistry and Molecular Biosciences, The University of Queensland, Brisbane 4072, Australia; z.tong@uq.net.au (Z.W.M.T.); a.karawita@uq.edu.au (A.C.K.); jane.sinclair@uq.net.au (J.E.S.); joylily_1@hotmail.com (L.Y.); kengyih.chew@uq.edu.au (K.Y.C.); 2 CSIRO, Australian Centre for Disease Preparedness, Health, and Biosecurity Business Unit, Geelong 3219, Australia; sue.lowther@csiro.au (S.L.); lee.trinidad@csiro.au (L.T.); arjun.challagulla@csiro.au (A.C.); michelle.baker@csiro.au (M.L.B.); 3 Department of Animal Science, University of California, Davis, CA 95616, USA; colin.kern@gmail.com (C.K.); hzhou@ucdavis.edu (H.Z.); 4 Department of Microbiology and Immunology, College of Veterinary Medicine, Cornell University, Ithaca, NY 14853, USA; kas24@cornell.edu; 5 Australian Infectious Diseases Research Centre, The University of Queensland, Brisbane 4072, Australia

**Keywords:** avian influenza, endothelial cells, chicken, duck, cytokine storm, H5N1

## Abstract

Highly pathogenic avian influenza viruses (HPAIVs) in gallinaceous poultry are associated with viral infection of the endothelium, the induction of a ‘cytokine storm, and severe disease. In contrast, in Pekin ducks, HPAIVs are rarely endothelial tropic, and a cytokine storm is not observed. To date, understanding these species-dependent differences in pathogenesis has been hampered by the absence of a pure culture of duck and chicken endothelial cells. Here, we use our recently established in vitro cultures of duck and chicken aortic endothelial cells to investigate species-dependent differences in the response of endothelial cells to HPAIV H5N1 infection. We demonstrate that chicken and duck endothelial cells display a different transcriptional response to HPAI H5N1 infection in vitro—with chickens displaying a more pro-inflammatory response to infection. As similar observations were recorded following in vitro stimulation with the viral mimetic polyI:C, these findings were not specific to an HPAIV H5N1 infection. However, similar species-dependent differences in the transcriptional response to polyI:C were not observed in avian fibroblasts. Taken together, these data demonstrate that chicken and duck endothelial cells display a different response to HPAIV H5N1 infection, and this may help account for the species-dependent differences observed in inflammation in vivo.

## 1. Introduction

Avian influenza A viruses (IAVs) represent an ongoing threat to the poultry industry, impacting animal health and causing major economic losses worldwide. Avian IAVs are categorised into low pathogenic avian influenza viruses (LPAIVs) and highly pathogenic avian influenza viruses (HPAIVs) based on their disease profile in gallinaceous poultry. In gallinaceous poultry, LPAIVs are often subclinical or cause only mild clinical symptoms. In contrast, HPAIVs in gallinaceous poultry can cause severe and fatal disease, often resulting in death within the first 24 h of infection. HPAIVs emerge in poultry following the insertion of a multi-basic cleavage site in the viral HA gene of low pathogenic H5 or H7 strains. Following their emergence in chickens, HPAIVs can spread to other avian or non-avian hosts (including humans) [1,2,3].

HPAIVs in chickens (*Gallus gallus*) primarily infect endothelial cells in a variety of different organs, including, but not limited to, the lung, heart, brain, and spleen [4]

Endothelial tropism by HPAIVs in chickens leads to a variety of different pathological consequences such as oedema, haemorrhaging, endothelial cell apoptosis, impaired thermoregulation, and disseminated intravascular coagulation [1]. In mice, despite the absence of widespread viral endothelial tropism, endothelial cells contribute to the uncontrolled, pro-inflammatory response to IAV. This ‘cytokine storm’ is also observed in chickens during HPAIV infections [5] and, given the pronounced endothelial cell tropism of HPAIVs in chickens, it is likely that poultry endothelial cells play a key role in this response [1]. However, due to the limited number of experimental systems available to study chicken endothelial cells, the role of chicken endothelial cells in the HPAI-associated cytokine storm remains to be defined.

In contrast to chickens, ducks have a markedly different host response to HPAIVs. The pathogenesis of HPAIV in ducks is dependent on factors such as the species of duck, the strain of HPAIV, and the age of the animal in question [2]. For example, compared to the mallard duck (*Anas platyrhynchos*), the wood duck (*Aix sponsa*) is significantly more susceptible to HPAI H5N1 [6]. However, despite such nuances, mallard ducks (and their domesticated derivative, Pekin ducks (*Anas platyrhynchos*)) typically only develop mild disease following HPAIV infection compared to chickens [1]. Importantly, in sharp contrast to chickens, in Pekin ducks, HPAIVs are not endothelial tropic and predominately infect other parenchymal cells [2]. In addition, a classical cytokine storm is not observed in ducks experimentally infected with H5N1 HPAIV [7].

These data raise the intriguing question as to what role endothelial cells play in the cytokine storm observed during HPAIV infection in chickens. This question has remained unanswered as, until recently, cell culture systems were not available for duck endothelial cells. However, we have recently shown that pure cultures of primary duck and chicken aortic endothelial cells can be obtained from both the bone marrow and aorta of embryonated eggs [7,8]. Consistent with in vivo observations, we have observed increased infection with A/*turkey*/*Turkey*/1/2005(H5N1) in chicken endothelial cells relative to those of ducks. However, in vitro endothelial cell infection of duck endothelial cells was still observed, suggesting that some additional factor was missing from the established in vitro culture system (e.g., another cell type or soluble factor), which may further limit infection in vivo. Here, we seek to use our previously characterised primary chicken and duck endothelial cells to understand the role of endothelial cells in species-dependent variations in the pathogenesis of HPAIVs.

## 2. Materials and Methods

### 2.1. Cell Culture

The use of embryos for cell culture was approved by the University of Queensland animal ethics committee (SCMB/002/18). Seventeen-day-old chicken and twenty-one-day-old Pekin duck embryonated eggs were purchased from Darling Downs Hatchery (Queensland, Australia). Primary aortic endothelial cells were cultured from the aortic arches of chicken and duck embryos as described previously using EGM-2MV medium (Lonza, Basel, Switzerland) with 10% FCS (Gibco, Waltham, MA, USA) [7,8]. Primary chicken and duck embryo fibroblasts were cultured as described previously [9,10]. Madin–Darby Canine Kidney (MDCK) cells were obtained from American Type Culture Collection (ATCC), and cultured in DMEM (Gibco, Waltham, MA, USA) supplemented with 10% FCS (Gibco, Waltham, MA, USA) and 1% penicillin/streptomycin (Gibco, Waltham, MA, USA). NCI-H441 cells (human lung epithelial cells) were obtained from ATCC and cultured in RPMI (Gibco, Waltham, MA, USA) medium supplemented with 10% FCS (Gibco) and 1% penicillin/streptomycin (Gibco). Primary human pulmonary microvascular endothelial cells were obtained from Sciencell (Carlsbad, CA, USA) and cultured in an endothelial cell growth medium (Sciencell, Carlsbad, CA, USA). All cell lines of mammalian origin were incubated at 37 °C 5% CO_2_ whilst all avian cells were cultured at 40 °C 5% CO_2_ unless otherwise stated.

### 2.2. Influenza Virus

A/chicken/Vietnam/0008/2004(H5N1) was amplified in embryonated chicken eggs as described previously [11]. All experiments using HPAIVs were performed under physical containment 3 (PC3) settings at the Australian Centre for Disease Preparedness (Geelong, Australia).

### 2.3. Viral Infection

Aortic endothelial cells were grown to confluency and were inoculated with 10^5^ PFU/mL of A/chicken/Vietnam/0008/2004 (H5N1) at a multiplicity of infection (MOI) of 1 for 60 min at 40 °C under physical containment 3 (PC3) settings. The inoculum was removed, and the cells were washed once with PBS before EGM-2MV media without FCS were added. The cells were incubated for 12 h. After removal of the supernatant, cellular RNA was extracted using the RNeasy Plus Kit (Qiagen, Hilden, Germany) according to the manufacturer’s instructions. Then, 3M of sodium acetate (NaOAc, pH 5.5) and 100% ethanol were subsequently added for RNA precipitation.

### 2.4. FITC-Labelled Acetylated Low-Density Lipoprotein (FITC-AcLDL) Uptake

Chicken, duck, and human endothelial cells of passages 10 and NCI-H441 cells of passage 11 were cultured to confluency and incubated with 0.33 µg/mL FITC-AcLDL (Invitrogen, Carlsbad, CA, USA) for 4 h. The cells were detached by washing thrice with PBS and incubating with 0.05% Trypsin-EDTA (Invitrogen, Waltham, MA, USA). The cells were resuspended in PBS containing 2% FCS and ac-LDL uptake was assessed using the BD Accuri^TM^ C6 Plus (BD Biosciences, Franklin Lakes, NJ, USA).

### 2.5. RNA Extraction, cDNA Synthesis and Reverse-Transcriptase Polymerase Chain Reaction (RT-PCR)

Endothelial cells from chickens and ducks were resuspended in 350 µL RNeasy lysis buffer (Qiagen, Hilden, Germany). RNA was extracted using the RNeasy Plus kit (Qiagen, Hilden, Germany) according to the manufacturer’s guidelines. Complementary DNA (cDNA) was synthesised with the High Capacity cDNA Reverse Transcription Kit (Applied Biosystems, Waltham, CA, USA) and random primers according to the manufacturer’s guidelines. RT-PCR was then conducted using the Phusion high-fidelity PCR kit (Biolabs, San Diego, CA, USA) and primers specific to avian von Willebrand factor (vWBF) and CD45 (Table S1). The samples were analysed on 1% agarose gels alongside with 1kb GeneRuler (Thermo Scientific, Waltham, MD, USA).

### 2.6. Flow Cytometry for Chicken CD45

Peripheral blood mononuclear cells (PBMCs) and aortic endothelial cells of passage 3 were thawed from liquid nitrogen, centrifuged at 400 g for 5 min and resuspended in PBS (Gibco) containing 2% FCS (Gibco) or 5 µg/mL mouse anti-chicken CD45 antibodies (Biorad, Hercules, CA, USA). The cells were incubated for 20 min before being centrifuged again at 1600 g for 5 min and resuspended in PBS (Gibco, Waltham, MA, USA) containing 2% FCS (Gibco) or 20 µg/mL Alexa Fluor 488 goat anti-mouse antibodies (Invitrogen, Waltham, MA, USA). The cells were incubated for 20 min before being centrifuged again at 1600 g for 5 min before resuspending in PBS (Gibco)) containing 2% FCS (Gibco). The cells were analysed with BD Accuri^TM^ C6 Plus (BD Biosciences, Franklin Lakes, NJ, USA).

### 2.7. Tube Formation Assay

Duck and chicken endothelial cells of passages 2–6 or MDCKs were grown to confluency and subsequently treated with 0.05% trypsin (Gibco). Approximately 7.5 × 10^3^ cells per cell type, diluted in EGM-2MV, were aliquoted into a well of a 96-plate coated with 50 µL of solidified Cultrex^®^ Basement Membrane ExtractType 2 (Trevigen, Gaithersburg, MD, USA). The plate was left to incubate at 37 °C 5% CO_2_ for 4 h. The media were removed, washed gently with 1% PBS, and the cells visualised under BX51 Upright Light Microscope (Olympus, Tokyo, Japan).

### 2.8. RNA Seq

RNA Seq was performed by Macrogen (Macrogen, Seoul, South Korea). Library preparation was performed using the Truseq stranded mRNA LT Sample Prep kit (Illumina, San Diego, CA, USA). All the libraries were barcoded and subsequently sequenced using the Novaseq 6000 (Illumina, San Diego, CA, USA) with Novaseq 6000 S4 Reagent Kit (Illumina, San Diego, CA, USA) as per the manufacturer’s instructions using within a single lane in a cell. RNAseq reads were separated into original samples based on the corresponding bar-code. RNAseq-based differential gene expression analysis was performed by assessing the quality of the raw reads using FastQC tool [12] before and after adaptor and quality trimming with TrimGalore version 0.6.2 (RRID: SCR_011847). RNAseq reads were then aligned to the Ensemble version 103 genomes and annotations of chicken (GRCg6a) and duck (CAU_duck1.0) using STAR 2.7.8a [13] with default parameters. Gene counts were determined using HTSeq version v0.13.5 [14] for individual libraries. The samples from each species were analysed for differential gene expression independently (duck endothelial cell experiment: three HPAIV VN/04(H5N1) infected groups and three mock-infected control groups; chicken endothelial cell experiment: four HPAIV VN/04(H5N1) infected groups and four mock-infected control groups). Once RNAseq read libraries were normalised for sequencing depth and RNA composition, differential gene expression analysis was performed using DESeq2 version 1.30.1 [15]. A q-value cut-off of 0.01 and log2 fold enrichment of 2 were used as the significance threshold (differential gene expression: mock vs. infected). KEGG pathway analysis [16] was performed using GAGE (R package) [17] and Pathview (R package) [18]. The raw RNAseq data are publicly available from the European Nucleotide Archive under the study number: PRJEB45405.

### 2.9. Immunofluorescence

Immunofluorescence was performed essentially as described previously [19]. Briefly, cells were fixed in 10% neutral buffered formalin, permeabilised with 0.1% IGEPAL CA-630 in PBS, and then stained using in-house rabbit polyclonal sera to the influenza A virus nucleoprotein.

### 2.10. PolyI:C Stimulation and Real-Time Quantitative PCR (RT-qPCR)

Cell cultures were grown in 24-well plates containing 5 × 10^4^ cells per well to confluency. The cell cultures were treated with 0.9% saline or 25 µg/mL PolyI:C (Sigma Aldrich, St. Louis, MO, USA). At various timepoints after the treatment, cell cultures were harvested by removing the media and resuspending 600 µL RNeasy Plus lysis buffer (Qiagen, Hilden, Germany) in each well. RNA was extracted from the cell lysates collected using the RNeasy Plus kit (Qiagen, Hilden, Germany) according to the manufacturer’s instructions. The RNA samples were then transcribed into cDNA using High-Capacity cDNA Reverse Transcription Kit (Applied Biosystems, Waltham, CA, USA). Samples were tested for glyceraldehyde 3-phosphate dehydrogenase (GADPH), interleukin 6 (IL-6) and IL-8 expression using RT-qPCR analysis (Table S1). SYBR Green PCR Master Mix (Applied Biosystems, Waltham, CA, USA) was used for SYBR qPCR analysis. qPCR conditions were conducted as per the manufacturer’s instructions for a 96-well reaction plate (Applied Biosystems, Waltham, CA, USA) and using QuantStudio 6 Flex Real-Time PCR System (Life Technologies, Carlsbad, CA, USA). GAPDH was used as a housekeeping gene, and relative gene expression was determined using the comparative cycle threshold method (ΔΔCt) relative to unstimulated control cells [20].

### 2.11. Statistical Analysis

Data were tested for normality using the Shapiro–Wilk test. Where data were normally distributed, data were analysed using a one-way ANOVA. Where data were not normally distributed, data were analysed using a Kruskal–Wallis test. Significance was set at *p* < 0.05. Statistical analyses were performed using GraphPad Prism.

## 3. Results

### 3.1. Chicken and Duck Endothelial Cells Can Be Cultured In Vitro

We have previously established a methodology to culture primary duck and chicken endothelial cells from the aorta and endothelial progenitor cells of embryonated eggs. Here, we once again confirmed that this methodology resulted in a pure population of primary duck and chicken endothelial cells (Figure S1). Endothelial cells were defined as cells able to take up FITC Ac-LDL, express vWBF, capable of tube formation and did not express CD45 or CD45+, and these cells formed <2% of the total population (Figure S1). In contrast, non-endothelial cells did not display the same gene expression profile (Figure S1), were unable to form tubes (Figure S2) and did not take up Ac-LDL (Figure S3).

### 3.2. Chicken and Duck Endothelial Cells Have a Different Transcriptional Response to HPAIV Infection

Having confirmed the identity and purity of chicken and duck endothelial cells, we next sought to determine if infection with HPAIV VN/04(H5N1) in vitro was associated with a specific gene expression profile. Accordingly, chicken and duck aortic endothelial cells were infected with HPAIV VN/04(H5N1), and 12 h post-infection cells were harvested for RNA extraction, followed by RNA sequencing and differential gene expression analysis. Endothelial cell infection was confirmed by the presence of viral RNA reads in infected chicken (mean 12.14% of library reads) and duck endothelial cells (mean 13.7% of library reads) and the absence of viral reads in mock-infected cells (0% library reads). Endothelial cell infection was further verified by immunofluorescent staining for influenza virus NP (Figure S4). In contrast to our previous findings with A/*turkey*/*Turkey*/1/2005(H5N1), here we did not observe differential infection of chicken and duck endothelial cells (Figure S4).

**Figure 1:**
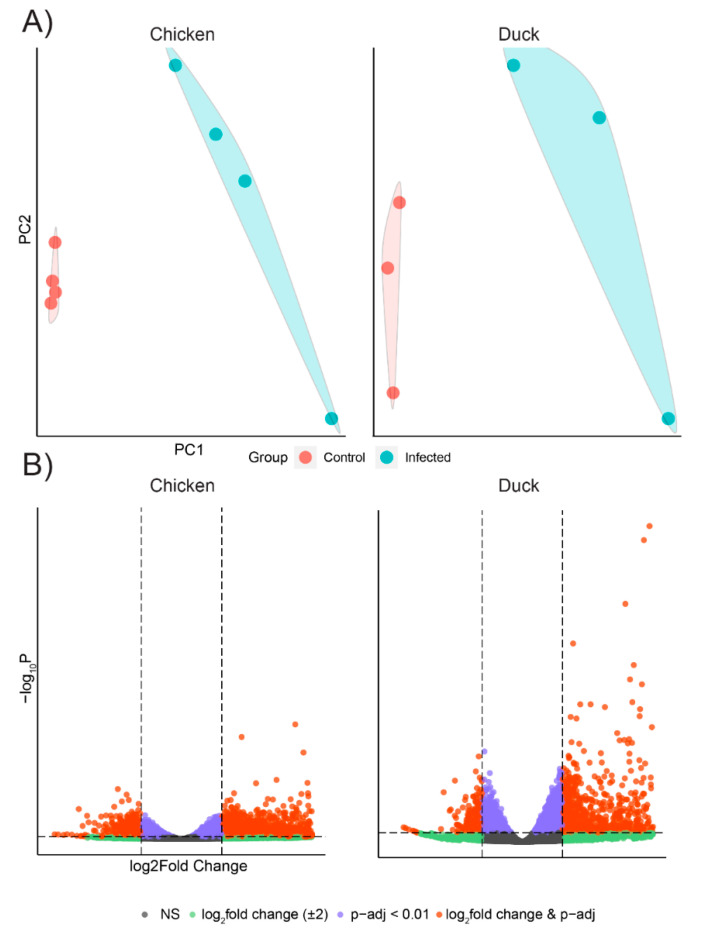
Chicken and duck endothelial cells have a different transcriptional response to HPAIV in vitro. RNA Seq analysis was performed 12 h post-infection on avian aortic endothelial cells inoculated with HPAI H5N1 (MOI 1). (**A**) Principal Component Analysis (PCA) plots of gene expressions based on RNAseq data. (**B**) Volcano plots showing differentially expressed genes between virus and mock-infected cells. DEGs (*q*-value = 0.01, log2fold enrichment of 2) are shown in orange. DEGs with a fold change enrichment of 2 (but *q*-value > 0.01) are shown in green. Genes that have *q*-value < 0.01 but not log2fold enrichment of 2 between the two treatment groups are shown in purple. NS = not significant (non-significant genes are shown in grey points).

**Figure 2:**
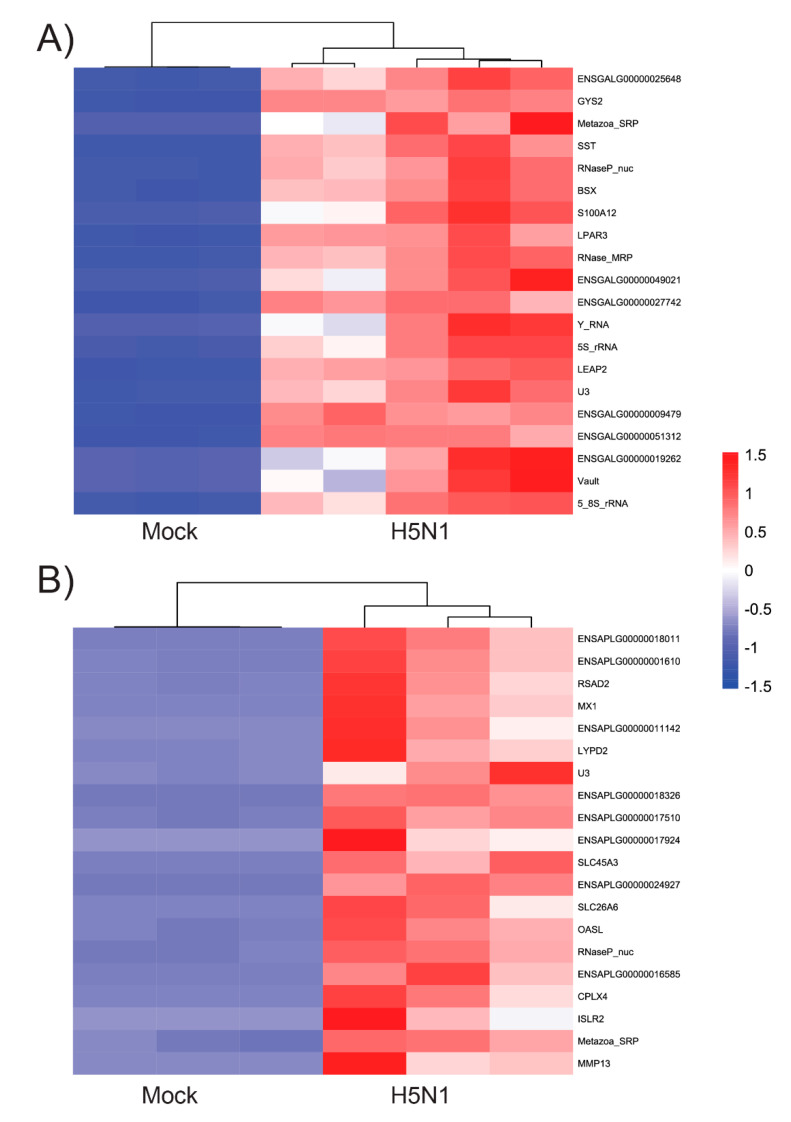
Heatmaps of the top 20 differentially expressed genes (the lowest q-value) of each species. Top differentially expressed genes of chicken (**A**) and duck (**B**) HPAIV VN/04(HPAI)-infected aortic endothelial cells against mock-infected cells.

Principle component analysis (PCA) of the top 1000 highly variable genes showed that the infected chicken and duck endothelial cells formed distinct clusters that were separate from those of their respective uninfected controls, although there was within-group variability amongst samples (Figure 1A). Genes that showed a log_2_-fold enrichment of 2 and q - value < 0.01 are considered differentially expressed genes (DEGs) and are shown in Figure 1B. We also plotted the top 20 of the most significantly expressed (the lowest q-value) genes of each species and performed hierarchical clustering based on ‘Euclidean’ distance for HPAIV VN/04(H5N1)-infected samples against mock-infected samples in a heatmap (Figure 2). We observed that the HPAIV VN/04(H5N1) infected and control groups form separate clusters similar to that of the PCA analysis. Moreover, compared to chicken cells, the top 20 genes of the heatmaps in HPAIV VN/04(H5N1)-infected duck cells involved well-known interferon-stimulated genes for antiviral defence ‘MX1′, ‘OASL’ and ‘RSAD2′ (Figure 2). We subsequently performed KEGG pathway analysis on chicken and duck differentially expressed genes. We elected to focus on enriched pro-inflammatory pathways as we aimed to determine if chicken endothelial cells could contribute to the aberrant cytokine production observed in vivo during a HPAI H5N1 virus infection. Numerous differentially expressed genes were associated with cytokine–cytokine interaction pathways (relative to mock infected cells) in infected chicken and duck cells (Table 1; Figures S5 and S6). Strikingly, in chickens, there were a number of genes upregulated in this pathway (Table 1). In contrast, in ducks, there were several genes downregulated in this pathway (Table 1). Of particular interest in this pathway is the expression of IL-6, IFNy, IL-18, IL-12, and IL-1B, key cytokines implicated in the pathogenesis of a cytokine storm [5,21]. These genes were all upregulated in infected chicken endothelial cells, whilst these genes were either not differentially expressed or downregulated in infected duck endothelial cells (Table 1). Together, these data are consistent with increased inflammation in infected chicken endothelial cells relative to those of ducks.

**Figure 3:**
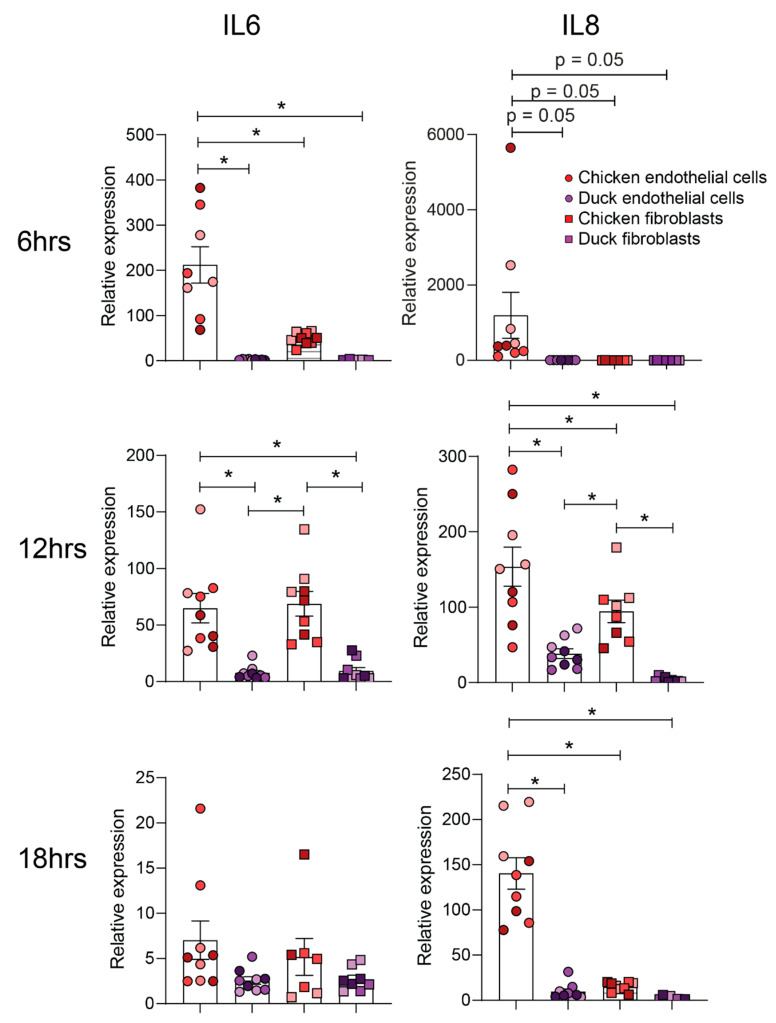
Cytokine response to polyI:C treatment in chicken and duck endothelial cells and fibroblasts. Avian endothelial and fibroblast cells were treated with either PBS or PBS containing 25 µg/mL PolyI:C (Sigma Aldrich). *IL-6* and *IL-8* gene expression were used as representatives for pro-inflammatory cytokine production and were analysed using the RT-qPCR analysis at different timepoints. Data are pooled (*n* = 9) from three independent experiments with experimental replicates shown in the same colour. Statistical significance is represented by asterisks (* = *p* < 0.05) Results are means ± SEM.

Next, we sought to identify whether the differential pro-inflammatory response of chicken and duck endothelial cells suggested by RNASeq data analysis was specific to HPAIV or whether it was a more generalised response to viral PAMPs. Accordingly, chicken and duck endothelial cells were stimulated with polyI:C and the expression of pro-inflammatory genes IL-6 and IL-8 was assessed at various timepoints post-stimulation. Strikingly, at 6, 12, and 18 h post-stimulation, chicken endothelial cells had higher relative levels of IL-6 and IL-8 compared to duck endothelial cells (Figure 3). The same experiments were repeated using chicken and duck fibroblasts to assess whether these species-dependent differences were specific to endothelial cells. Only at 12 h post-stimulation was a significant difference observed in IL-6 and IL-8 expression between chicken and duck fibroblasts (Figure 3). Moreover, at 6, 12, and 18 h post-stimulation, chicken endothelial cells had significantly higher relative levels of IL-6 and/or IL-8 compared to both chicken and duck fibroblasts (Figure 3). These data suggest that whilst there may be inherent differences in the pro-inflammatory response to polyI:C in multiple different duck and chicken cells, this effect is more pronounced in endothelial cells.

## 4. Discussion

Chickens and ducks have a markedly different response to HPAIV. In contrast to ducks, HPAIV in chickens induces a cytokine storm and primarily infects the endothelium. However, until recently, culture methods did not exist for chicken and duck endothelial cells. This significantly impaired our ability to assess, in detail, the role of endothelial cells in the cytokine storm observed in chickens.

Here, we used a recently validated method of avian endothelial cell culture to show that aortic chicken endothelial cells mount a more robust pro-inflammatory response to HPAIV compared to aortic duck endothelial cells. In contrast to our previous studies, performed using another strain of H5N1, we observed equivalent viral infection in duck and chicken endothelial cells. This contracts with the in vivo situation and suggests that some additional in vivo factor was missing from the established in vitro culture system. However, the equivalent in vitro infections suggest that any transcriptional differences observed resulted from intrinsic differences in the cellular response to viral infection, rather than the absence of viral infection in one species. The observed species-dependent differential transcriptional response was characterised by an upregulation of pro-inflammatory cytokines in chickens and a downregulation of select pro-inflammatory cytokines in ducks. An augmented pro-inflammatory response in chicken cells compared to those of ducks is consistent with previous studies both in vitro and in vivo [21,22,23]. However, these data are the first to provide evidence that endothelial cells contribute to these species-dependent differences.

Importantly, in the present study, we showed that a more pronounced pro-inflammatory response in chicken endothelial cells was not specific to HPAIV infection as a similar response was observed following polyI:C stimulation. Whilst polyI:C also triggered a pro-inflammatory response in chicken and duck fibroblasts, this was not as pronounced as the pro-inflammatory response observed in chicken endothelial cells. This is consistent with mammalian studies, where endothelial cells have been identified as the key drivers of the cytokine storm during severe IAV infections [24].

There are inherent limitations to using a reductionist in vitro system to understand the pathogenesis of an in vivo infection. Future studies would benefit from the co-culture of additional cell types alongside endothelial cells to better reflect the in vivo situation. Nevertheless, the data presented here emphasise the need, even in non-model species, to study multiple different cell types to properly elucidate viral pathogenesis.

## 5. Conclusions

In this study, we have provided evidence that compared to primary duck endothelial cells, primary chicken endothelial cells exhibit a heightened pro-inflammatory response to both polyI:C and HPAI viruses. This suggests a role of endothelial cells in the cytokine storm detected in chickens in response to HPAI viruses. These data further emphasise the utility of primary cell cultures in studying species-dependent differences in viral pathogenesis. 

## Figures and Tables

**Table 1:** Cytokine–cytokine interaction pathways in chickens and ducks.

	Upregulated in Duck (Total Number of Genes)	Upregulated in Chicken (Total Number of Genes)	Downregulated in Duck (Total Number of Genes)	Downregulated in Chicken (Total Number of Genes)
Chemokines	CCL21, CCL4, CCL41, CCL4L2, CCL5, CCL28, CCR8, CCR9 (8)	CCL19, CCL21, CCL4, CCL41, CCL42, CCL17, CCL5, CCR8, CCR9, CCR4, CCR5 (11)	CCR11, CCR5, CXCL13, CXCR7 (4)	CCR7 (1)
The class I helical cytokines	IL2RB, IL2RG, IL7R, IL21R, IL5RA, CSF2RA, GHR, PRLR, TPO, MPL, CSF3, IL6ST, IL12RB2, IL23R, IL13RA, LIFR, (16)	IL2RA, IL2RB, IL2RG, IL9R, IL21R, CSF2RB, CSF2RA, IL13RA2, PRL, PRLR, GHR, TPO, CSF3, IL6, IL11, IL12, IL6ST, IL12, IL12RB2, IL35, CLCF1, CNTF, (22)	IL9R, IL15RA, CSF2RB, IL13RA2, CSFR, LEPR, IL6R, IL6, OSMR (9)	IL4R, IL13RA1, GH1, GH2, CSF3R, IL6R (6)
The class II helical cytokines	IL10RB, IL20RA, IL20, IL28A, IL28B (5)	IL10, IL20, IL20RA, IL22, IL10RB (5)	IL22RA1, IFNGR1 (2)	
Interferon family	IFNAR2, IFNGR2 (2)	IFNB1, IFNAR2, IFNG, IFNGR1, IFNGR2 (5)		
IL-1-like cytokines	IL1RAP, IL1RL2, IL18R1, IL18RAP, ST2 (5)	IL1B, IL1RAP, IL1R2, IL1R2, IL18R1, IL18, IL18RAP, ST2 (8)	IL1R1, IL1R2, IL18 (3)	IL1R1 (1)
IL-17-like cytokines	IL17RA, IL17B, IL17C, IL17D (4)	IL17F, IL17B (2)	IL17RE (1)	
Non-classified	IL16, IL34, CSF1R (3)	IL16, CD4, IL34, CSF1, CSF1R (5)	CSF1 (1)	
TNF Family	DCR3, FASLG, FAS, VEGI, DR6, EDA, CD30L, CD40LG, CD30, 4-1BB, OX40L, Ox40G, GITRL, GITR, BCMA, BAFF (16)	FASLG, VEG1, DR4, DR5, DR6, EDA, EDAR, NGF, NGFR, RANKL, OPG, CD30, CD40LG, GITR, BAFF, TACI, RELT (17)	TNFR1, TNFR2, XEDAR, CD40, TROY (5)	TNFR2, DCR3, FAS, RANK, CD40 (5)
TGF-beta family	TGFBR2, ACVRL1, TGFBR2, GDF15, GDF2, ACVR2B, GDF10, GDF11, MSTN, INHBA, INHBB, GDF1, AMH, BMPR1B, BMP6, BMP8 (16)	GDF15, GDF11m INHBA, INHBB, GDF1, ACVR1C, GDF9, AMH, AMHR2, BMP4, BMPR1B, BMP5, BMP6 (13)	TGFB3, TGFBR1, ACVR2, BMPR2, BMP2, BMP4, GDF6, GDF7, BMP15, BMP5, ACVR2A (11)	TGFBR1, TGFBR2, ACVR2A, BMPR2, INHBC, BMPR1A, ACVR2B (7)

## Data Availability

The raw RNAseq data are publicly available from the European Nucleotide Archive under the study number: PRJEB45405.

## Supplementary Materials

The following are available online at https://www.mdpi.com/article/10.3390/genes12060901/s1, Figure S1: Confirmation of endothelial phenotype in chicken and duck cells, Figure S2: MDCKs do not form tubes in a tube-forming assay. Figure S3: LDL uptake by human epithelial cells and endothelial cells. Figure S4: Chicken and duck endothelial cells are infected with HPAIV in vitro. Figure S5: Significant enrichment of cytokine-cytokine receptor interaction pathways in chicken endothelial cells following infection with HPAIV H5N1, Figure S6: Significant enrichment of cytokine-cytokine receptor interaction pathways in duck endothelial cells following infection with HPAIV H5N1, Table S1: Primers used in this study.
