# Supplementary material for: Primary Chicken and Duck Endothelial Cells Display a Differential Response to Infection with Highly Pathogenic Avian Influenza Virus
Source: Genes (Basel). 2021 Jun 10;12(6):901. doi: 10.3390/genes12060901 (PMC8230508; doi:10.3390/genes12060901)
Supplement: Supplementary file 1 [file genes-12-00901-s001.zip › genes-1236809-supplementary.pdf]

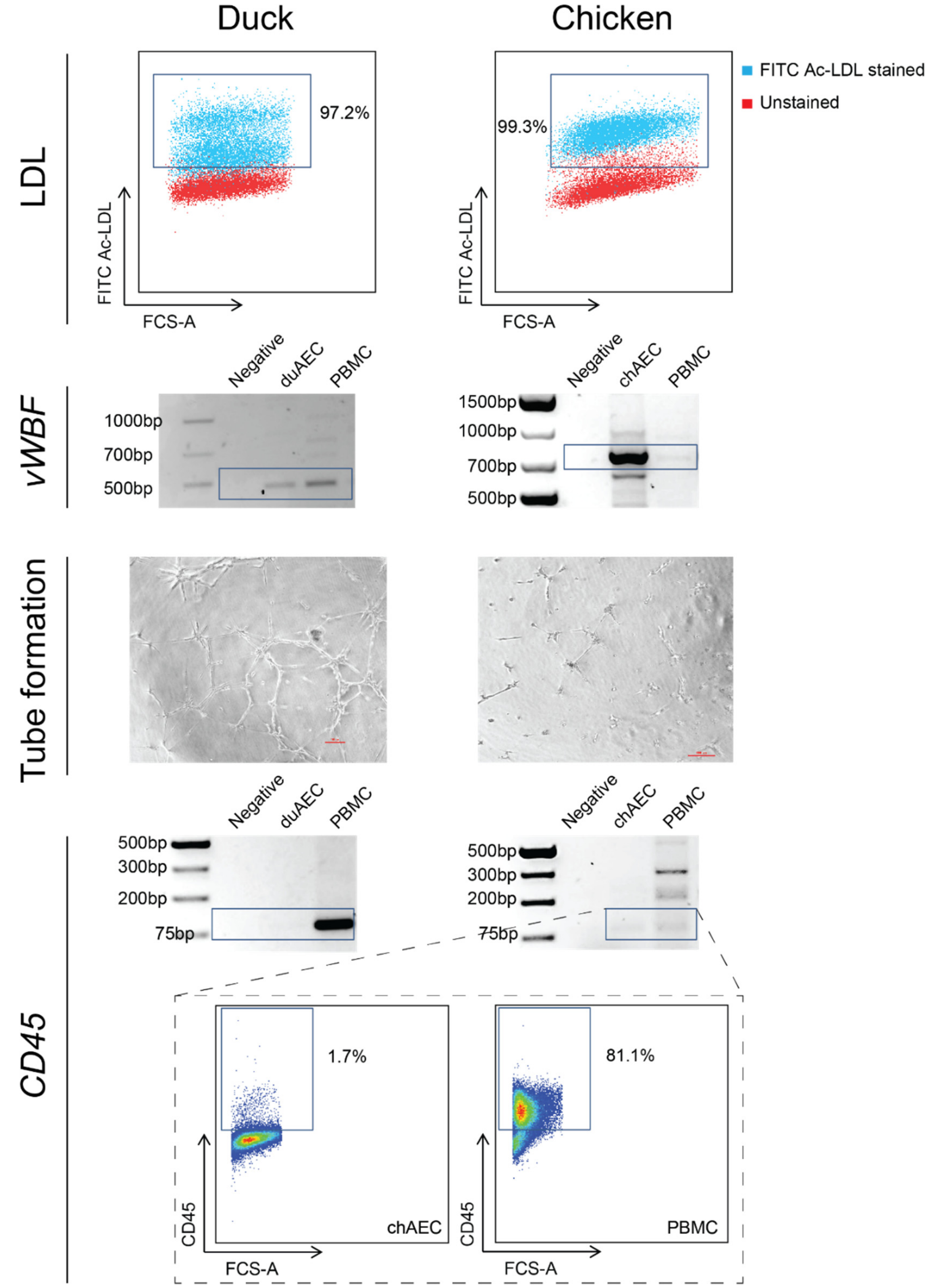

Supplementary Figure 1. Confirmation of endothelial phenotype in chicken and duck cells. Endothelial cells isolated from the aortic arches of chickens and ducks were used for a series of characterisation tests to confirm the endothelial phenotype. *Low Density Lipoprotein (LDL)*: Chicken and duck endothelial cells were incubated with 0.33µg/ml FITC-AcLDL for 4 hours at 40 °C and the cells were quantified using FACS analysis. Representative overlay FACS plots are shown and the percentage of AcLDL+ cells are indicated. *von Willibrand Factor (vWF)*: Expression of vWBF in aortic endothelial cells (AECs) and peripheral blood mononuclear cells (PBMCs). *Tube formation*: Avian cells were left to incubate at 40°C 5% CO_2 for 4 hours in wells coated with 50µl of solidified Cultrex® Basement Membrane ExtractType 2 (Trevigen, US). Tube formation is indicated. *CD45 expression*: Expression of

vWBF in aortic endothelial cells (AECs) and peripheral blood mononuclear cells (PBMCs). The percentage of CD45+ positive cells in chicken samples was then assessed by flow cytometry. Representative FACS plots are shown and the percentage

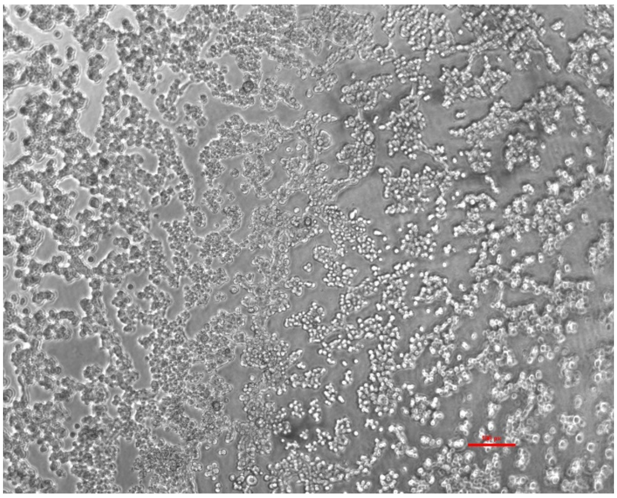

Supplementary Figure 2: MDCKs do not form tubes in a tube-forming assay. A representative image of MDCK cells that were left to incubate at 37°C 5% CO_2 for 4 hours in wells coated with 50µl of solidified Cultrex® Basement Membrane ExtractType 2 (Trevigen, US).

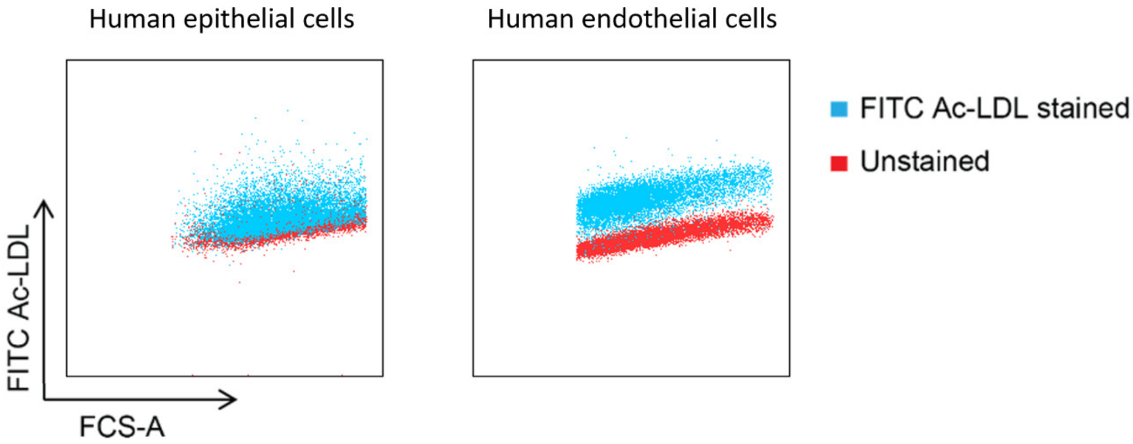

Supplementary Figure 3: LDL uptake by human epithelial cell and endothelial cells. Cells were incubated with 0.33µg/ml FITC-AcLDL for 4 hours and quantified using FACS analysis. Representative overlay FACS plots are shown.

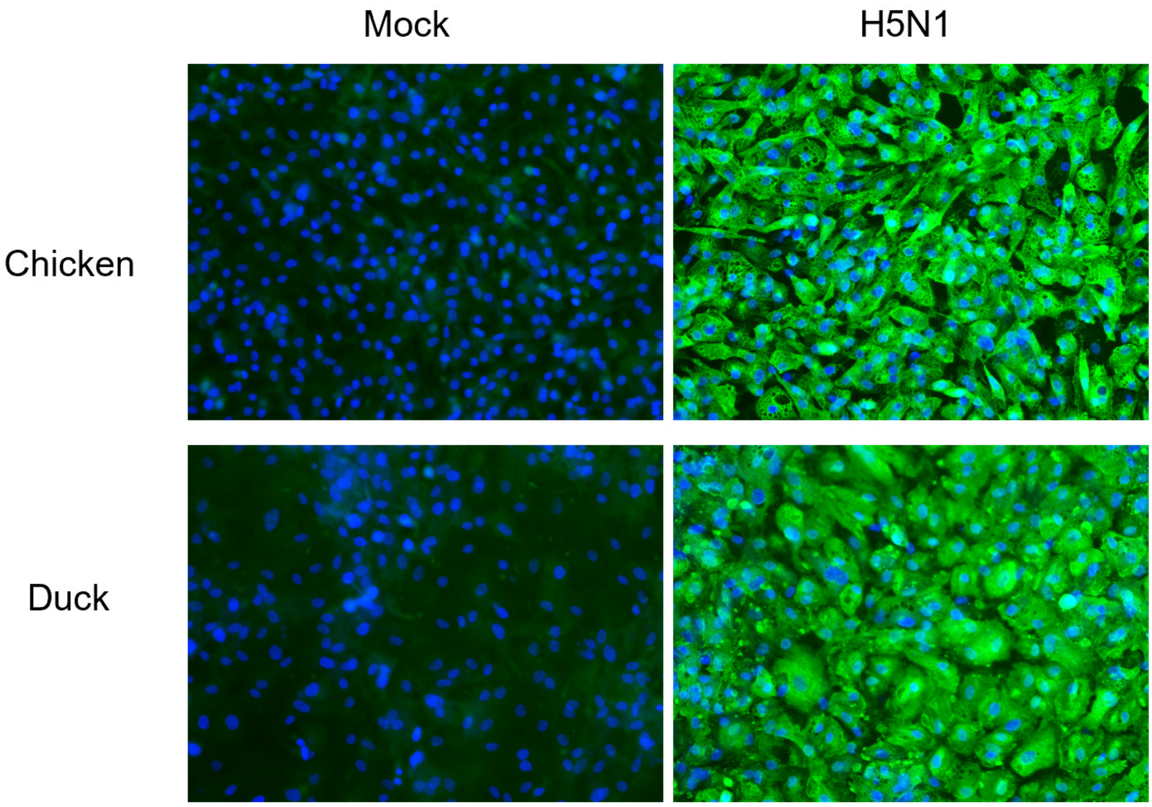

Supplementary Figure 4: Chicken and duck endothelial cells are infected with HPAIV *in vitro*. Representative images are shown for duck and chicken endothelial cells stained for influenza virus nuclear protein (green) and DAPI (blue).

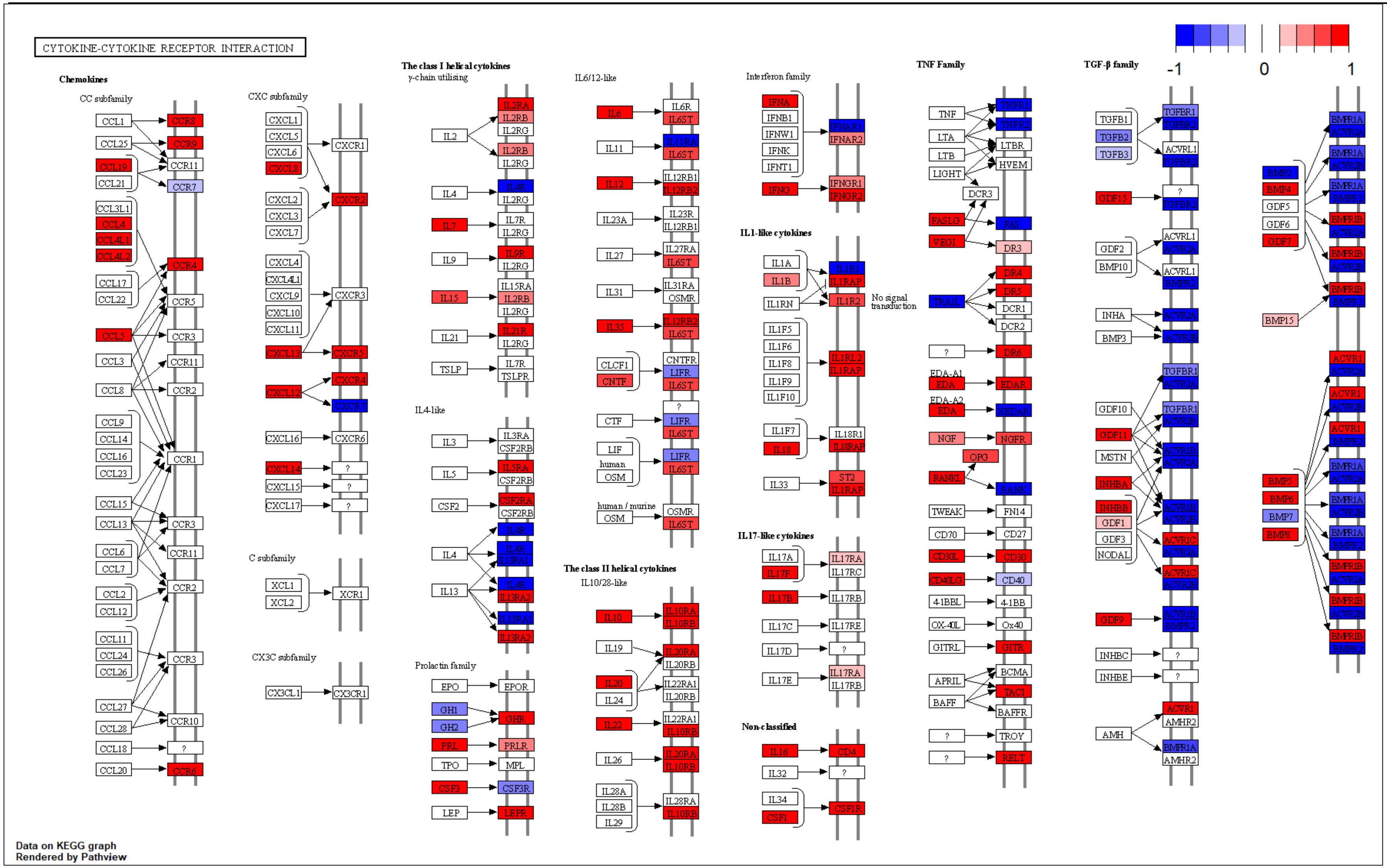

Supplementary Figure 5: Significant enrichment of cytokine-cytokine receptor interaction pathways in chicken endothelial cells following infection with HPAIV H5N1. Upregulated genes (relative to uninfected cells) are shown in red whilst downregulated genes (relative to uninfected cells) are shown in blue.

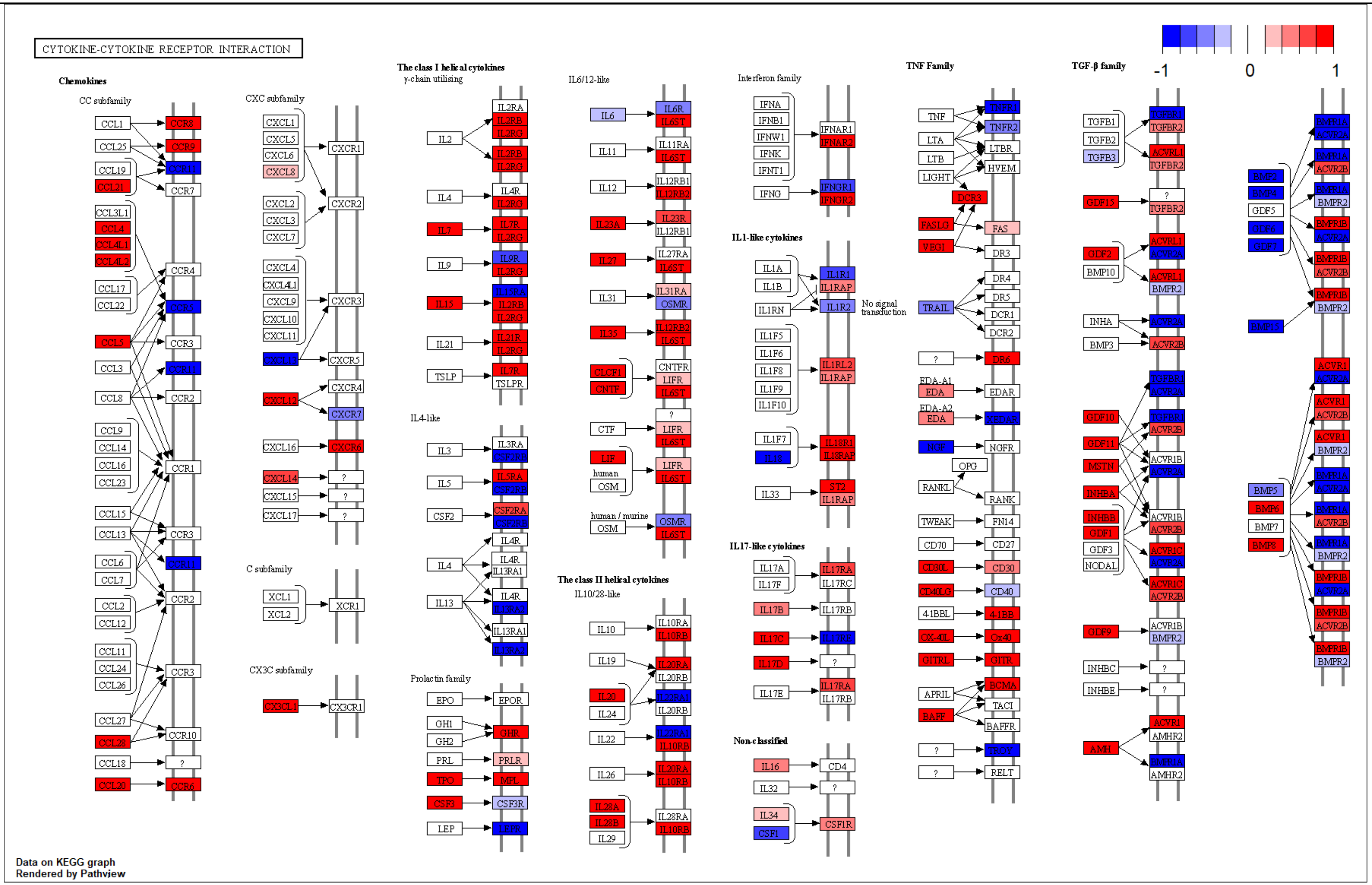

Supplementary Figure 6: Significant enrichment of cytokine-cytokine receptor interaction pathways in duck endothelial cells following infection with HPAIV H5N1. Upregulated genes (relative to uninfected cells) are shown in red whilst downregulated genes (relative to uninfected cells) are shown in blue.

genes

MDPI

Supplementary Table 1: Primers used in this study

Gene	Species	Sequence
vWBF	Chicken	5′-GCCAATGACTTCATG-3′
		5′-GCCACAGTCATTGGTG-3′
	Duck	5′-ACCACATGTTAGTGAGGAAC-3′
		5′- CTTGGTAGGGTATGCTTCTC-3′
CD45	Chicken	5′- CAACATCCTTGCACAACACC-3′
		5′-CTCTTCCCATCTTCCAGCAG-3′
	Duck	5′-ATTGCCAGTATCTACCCTGGC-3′
		5′-TGTTGAGCTTTCTGTTCCCT-3′
GAPDH	Chicken	5′-GGTGCTAAGCGTGTTATCATCTCA-3′
		5′-CATGGTTGACACCCATCACAA-3′
	Duck	5′- GCCTCTTGCACCACCAACT-3′
		5′-GGCATGGACAGTGGTCATAA-3′
IL6	Chicken	5′-GCGAGAACAGCATGGAGATG-3′
		5′-GTAGGTCTGAAAGGCGAACAG-3′
	Duck	5′-GCAACGACGATAAGGCAGATG-3′
		5′-TCTTATCCGATTTCAGCTTTGTGA-3′
IL8	Chicken	5′-CTGCGGTGCCAGTGCATTAG-3′
		5′-AGCACACCTCTCTTCCATCC-3′
	Duck	5′-AGGACAACAGAGAGGTGTGCTTG-3′
		5′-GCCTTTACGATCCGCTGTACC-3′

Author Contributions: Conceptualization, MLB & KRS. Data acquisition: ZWMT, JES, SL, LT, AC, KAS Data analysis: ACK, CK, HZ, LY, KYC, MLB, KRS writing—original draft preparation, ZWMT, ACK, KRS supervision, MLB, KRS.; project administration, KRS.; funding acquisition, KRS. All authors have read and agreed to the published version of the manuscript.

Funding: AC was funded by CSIRO via a Research Office postdoctoral fellowship. KAS was supported by the CSIRO Research Office via the Distinguished Visiting Researcher funding scheme. KRS was funded by the Australian Research Council (Fellowship DE180100512). This work was funded by the Australian Department of Agriculture and Water Resources (CSIRO Science and Innovation Award).

Institutional Review Board Statement: The use of embryos for cell culture was approved by the University of Queensland animal ethics committee (SCMB/002/18).

Data Availability Statement: The raw RNAseq data are publicly available from European nucleotide archive under the study number : PRJEB45405.

Conflicts of Interest: The authors declare no conflict of interest.
